# Financial toxicity of patients with lung cancer in China: Results from a National Survey Study

**DOI:** 10.1002/cam4.5244

**Published:** 2022-09-21

**Authors:** Meicen Liu, Dengmin Huang, Yuanli Liu

**Affiliations:** ^1^ School of Health Policy and Management Chinese Academy of Medical Sciences and Peking Union Medical College Beijing People's Republic of China

**Keywords:** China, financial toxicity, lung cancer, National survey

## Abstract

**Objective:**

To develop a comprehensive understanding of financial toxicity (FT) among patients with lung cancer in China and the major factors affecting FT.

**Methods:**

Drawing from a national cross‐sectional survey, which used the validated comprehensive score for financial toxicity (COST) questionnaire, we estimated the prevalence and degree of FT. Patient coping actions were investigated. Pearson's chi tests and multinomial logistic regression were used to evaluate the predictors of FT in patients with lung cancer.

**Results:**

The median score of FT was 20 (scored on a range of 0–44, with lower scores indicating more severe toxicity). Altogether, 77% of the sample patients had FT (COST <26), 54.5% had mild FT (COST 14–25), and 22.5% had moderate and severe FT (COST 0–13). Living in the less‐developed western region of China, being male, having a lower educational level, lower annual family income, and advanced stage or worse self‐reported health status were significantly related to higher FT than their counterparts (*p* < 0.05). Patients with higher FT tended to have a lower level of medical compliance, a higher risk of incurring debts, and reduced living expenditures relative to those with lower FT.

**Conclusion:**

Despite China's remarkable progress in the past two decades with regard to Universal Healthcare Coverage, FT still presents a serious challenge for patients with lung cancer. Keen attention must be paid to reducing the disproportionate high financial risks of patients with low socioeconomic status.

## Graphical abstract

In this national survey, a relatively high prevalence of FT was observed in patients with lung cancer in China. Patients with high FT are more likely to use coping actions. Keen attention must be paid to reducing the disproportionate high financial risks of those patients with low SES.

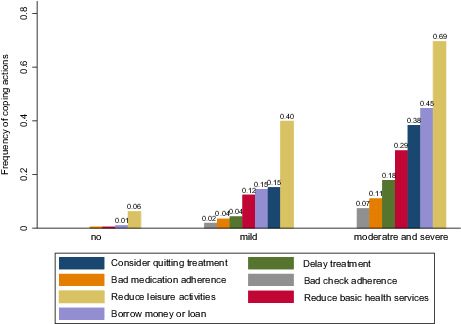

## 1 INTRODUCTION

Financial toxicity (FT) refers to the subjective financial distress and objective financial burden of medical care expenditures.^1^ An increasing amount of attention has been paid to FT in patients with cancer, in large part due to the rising costs of cancer diagnosis and treatment.^2^ Previous studies of FT in cancer patients have examined the prevalence of FT among subgroups of patients with different cancer types,^3^, ^4^, ^5^, ^6^ cancer stages,^5^, ^7^, ^8^ treatment schemes,^5^, ^9^ healthcare coverage systems,^10^, ^11^, ^12^ or other subgroups as well as related factors of FT. These studies indicated disparities in prevalence and related factors among subgroups. Some studies have found that FT led to decreased medication adherence, delayed treatment, poorer quality of life, and difficulties in returning to work.^13^, ^14^, ^15^ Recently, qualitative and quantitative strategic studies have been conducted to find ways of reducing FT.^16^ Some strategies have been adopted by clinical oncology guidelines or decision makers, such as shared decision making for costs associated with different treatment regimens.^17^

This study focused on FT in patients with lung cancer in China for several reasons. Lung cancer is a prominent cause of cancer incidence and mortality worldwide. In 2015, China had an estimated 733,300 newly diagnosed and 610,200 death cases.^18^, ^19^ Additionally, lung cancer is the most expensive cancer to treat and imposes an enormous financial burden on individuals and families.^20^, ^21^, ^22^ Previous studies among patients with lung cancer indicated negative consequences and identified some predictors of FT. For example, Hazell et al. reported that FT was associated with poorer quality of life and that work status was a predictor in the United States.^23^ Ezeife et al. found that age, work, insurance, aid, clinical trials, and out‐of‐pocket costs were predictors in Canada.^12^ Friedes et al. found that risk factors of FT at diagnosis were different from risk factors at the 6‐month follow‐up, indicating that disease duration may be a predictor.^24^ However, only one study was conducted in the western region of China, and it found that age, savings, and work status were risk factors for FT among advanced lung cancer patients.^25^ In summary, existing studies are limited in their scope to help inform on FT and coping actions among patients with lung cancer in large countries such as China.

This study used data from a national survey to better characterize FT among patients with lung cancer including (1) prevalence of FT categorized as mild, moderate, and severe; (2) major predictors of FT including demographic, economical, and clinical characteristics; and (3) patient coping actions in response to FT.

## 2 METHODS

### 2.1 Study design

This study was based on the cross‐sectional China National Patient Survey (CNPS), which has been sampling over 200 public tertiary hospitals annually since 2015.^26^, ^27^ The 2021 CNPS, which was conducted for 3 months from January to March 2021, sampled a total of 143 tertiary hospitals (general, traditional Chinese medicine, maternal, and pediatric), 41 psychiatric hospitals, and 33 tertiary cancer hospitals from 31 provinces. These hospitals represent China's national and regional referral centers and regularly offer treatments for a large number of patients with severe and complicated diseases.

### 2.2 Participants

At least 150 inpatients were continuously recruited from each hospital between January and March 2021. Each patient was interviewed by trained investigators. All patients were nearing discharge at the time of the interview, and those who agreed to participate in the study were asked to complete an electronic questionnaire. The investigators assisted patients who were unable to complete the questionnaire on their own.

This study was approved by the Ethics Committee of the Institute of Medical Biology of Chinese Academy of Medical Sciences (IPB‐2020‐23) and all patients provided informed consent for their participation in the study.

**FIGURE 1:**
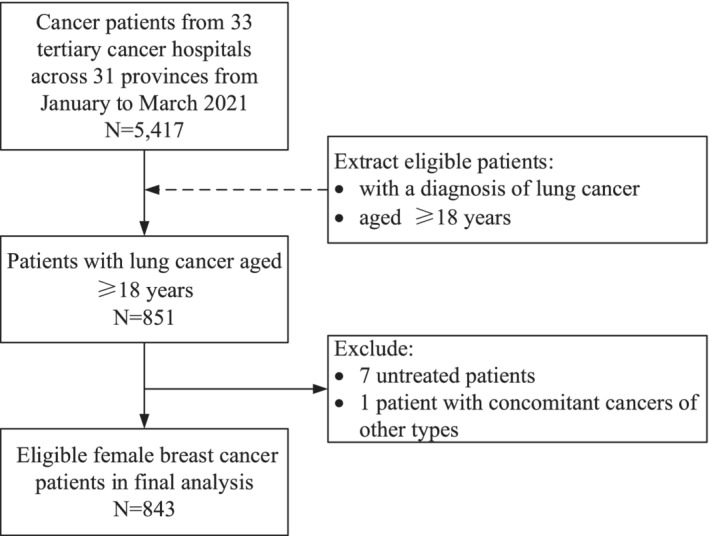
Flowchart describing the selection process for included participants

This study focused on patients with lung cancer of 33 sampled tertiary cancer hospitals. A total of 5417 patients were surveyed at these hospitals. Patients aged ≥18 years, with a diagnosis of lung cancer, were selected for further analysis. From this cohort, eight patients (seven patients who did not undergo treatment during hospitalization and one patient with concomitant cancers of other types) were excluded. The final analysis included 843 patients with lung cancer (Figure 1).

### 2.3 Variables and outcomes

The survey included questions about patients' demographics (residence region, residence type, gender, age, marital status, and education), economic information (work, annual family income, medical insurance, and medical assistance), clinical information (cancer duration, cancer stage, self‐reported health status, and length of stay), and validated measures of FT. FT was measured using the comprehensive score for financial toxicity (COST) tool, which has been validated and used internationally and in China; it consists of 12 items rated with a 5‐point Likert scale.^28^, ^29^, ^30^ The total score of the COST ranged from 0 to 44 points. Lower scores indicated higher levels of FT and worse financial conditions. The original version of the COST tool and the scoring guideline are available online (https://www.facit.org/measures/FACIT‐COST).

We used COST <26 on a scale of 0–44 as a threshold for screening positive for FT. We also categorized the degree of FT based on a proposed grading scale: no FT (COST ≥26), mild FT (COST 14–25), moderate and severe FT (COST 0–13).^31^, ^32^

The coping actions were collected using a survey question, “have you ever taken the following actions due to financial difficulties,” followed by seven items with “yes” or “no” responses:

1. once considered quitting treatment,
2. have delayed treatment for more than 7 days.
3. have failed to take medicine as instructed,
4. have failed to attend medical visits as instructed,
5. have reduced spending on leisure activities, such as shopping or traveling,
6. have reduced spending on basic health services, such as clinic visits or vaccinations, and
7. have borrowed money or acquired a loan due to illness.

### 2.4 Statistical analysis

Descriptive statistics were used to summarize FT, coping actions, and characterize the patient population. Pearson's chi‐square tests and multinomial logistic regression were used to evaluate associations between patient characteristics and different degrees of FT. Multinomial logistic regression was used with FT as a nominal three‐category variable, namely (a) no FT, (b) mild FT, and (c) moderate and severe FT, with the “no FT” category being the reference value. Ordered logistic regression was not used because the parallel assumption was not accepted.

All statistical analyses were conducted with Stata/SE 15.0 software (Stata Corp LP, College Station, TX, USA). A two‐tailed *p* value of <0.05 was considered statistically significant.

## 3 RESULTS

### 3.1 Prevalence of financial toxicity: mild, moderate, and severe

In 843 patients with lung cancer, the mean score of FT was 20.32 (standard deviation 9.514), and the median score was 20 (interquartile range 14–25). There were 194 (23.01%) patients with no FT, 459 (54.45%) patients with mild FT, and 190 (22.54%) patients with moderate and severe FT.

### 3.2 Patient characteristics and financial toxicity

Of the 843 participants, 487 (57.77%) were living in urban regions, 558 (66.19%) were male, 219 (32.99%) were aged older than 65 years, 165 (19.57%) had cancer stage 3, and 254 (30.13%) had cancer stage 4 (Table 1). The difference among the three FT groups was significant after stratifying patients into groups including residence region, residence type, gender, education, work, income, medical assistance, cancer type, and self‐reported health status (*p* < 0.05).

**TABLE 1:** Patient characteristics

Patient characteristics	All patients (*N* = 843)	No FT (*N* = 194)	Mild FT (*N* = 459)	Moderate and severe FT (*N* = 190)	*χ* ^2^	*p* value
Demographic characteristics						
Residence region						
Eastern	332 (39.38)	97 (29.22)	169 (50.90)	66 (19.88)	**15.498**	**0.004**
Central	289 (34.28)	63 (21.80)	161 (55.71)	65 (22.49)		
Western	222 (26.33)	34 (15.32)	129 (58.11)	59 (26.58)		
Residence type						
Rural	356 (42.23)	59 (16.57)	196 (55.06)	101 (28.37)	**20.448**	**<0.001**
Urban	487 (57.77)	135 (27.72)	263 (54.00)	89 (18.28)		
Gender						
Female	285 (33.81)	85 (29.82)	135 (47.37)	65 (22.81)	**12.658**	**0.002**
Male	558 (66.19)	109 (19.53)	324 (58.06)	125 (22.40)		
Age (years)						
<55	257 (5.93)	58 (22.57)	130 (50.58)	69 (26.85)	6.312	0.177
55–65	367 (15.30)	84 (22.89)	200 (54.50)	83 (22.62)		
>65	219 (32.99)	52 (23.74)	129 (58.90)	38 (17.35)		
Marital status						
Married	791 (93.83)	186 (23.51)	433 (54.74)	172 (21.74)	5.189	0.075
Single/divorced/widowed	52 (6.17)	8 (15.38)	26 (50.00)	18 (34.62)		
Education						
College	190 (22.54)	65 (34.21)	96 (50.53)	29 (15.26)	**22.621**	**0.001**
High school	234 (27.76)	48 (20.51)	122 (52.14)	64 (27.35)		
Junior school	252 (29.89)	51 (20.24)	146 (57.94)	55 (21.83)		
Primary School or less	167 (19.81)	30 (17.96)	95 (56.89)	42 (25.15)		
Economic characteristics						
Work						
Public institutions	152 (18.03)	42 (27.63)	85 (55.92)	25 (16.45)	**31.169**	**0.001**
Private or individual institutions	144 (17.08)	33 (22.92)	83 (57.64)	28 (19.44)		
Farmer	236 (28.00)	40 (16.95)	120 (50.85)	76 (32.20)		
Retired	224 (26.57)	65 (29.02)	122 (54.46)	37 (16.52)		
Unemployed	65 (7.71)	8 (12.31)	39 (60.00)	18 (27.69)		
Other	22 (2.61)	6 (27.27)	10 (45.45)	6 (27.27)		
Annual family income (1000 yuan)						
<30	256 (30.37)	32 (12.50)	141 (55.08)	83 (32.42)	**92.891**	**<0.001**
30–60	295 (34.99)	44 (14.92)	175 (59.32)	76 (25.76)		
>60	292 (34.64)	118 (40.41)	143 (48.97)	31 (10.62)		
Medical insurance						
UEBMI	315 (37.37)	80 (25.40)	183 (58.10)	52 (16.51)	**34.865**	**<0.001**
URBMI	471 (55.87)	87 (18.47)	255 (54.14)	129 (27.39)		
Other	57 (6.76)	27 (47.37)	21 (36.84)	9 (15.79)		
Medical assistance^†^						
Yes	112 (13.29)	25 (22.32)	58 (51.79)	29 (25.89)	0.840	0.657
No	731 (86.71)	169 (23.12)	401 (54.86)	161 (22.02)		
Clinical characteristics						
Cancer duration (years)						
<1	392 (46.50)	98 (25.00)	205 (52.30)	89 (22.70)	2.199	0.699
1–2	208 (24.67)	44 (21.15)	115 (55.29)	49 (23.56)		
≥2	243 (28.83)	52 (21.40)	139 (57.20)	52 (21.40)		
Cancer stage						
0–1	147 (17.44)	48 (32.65)	77 (52.38)	22 (14.97)	**22.814**	**0.004**
2	77 (9.13)	14 (18.18)	53 (68.83)	10 (12.99)		
3	165 (19.57)	35 (21.21)	93 (56.36)	37 (22.42)		
4	254 (30.13)	49 (19.29)	136 (53.54)	69 (27.17)		
NA	200 (23.72)	48 (24.00)	100 (50.00)	52 (26.00)		
Self‐reported health status						
Worse	293 (34.76)	47 (16.04)	156 (53.24)	90 (30.72)	**26.028**	**<0.001**
Moderate	323 (38.32)	78 (24.15)	182 (56.35)	63 (19.50)		
Better	227 (26.93)	69 (30.40)	121 (53.30)	37 (16.30)		
Length of stay						
≦7 days	442 (52.43)	93 (21.04)	253 (57.24)	96 (21.72)	3.177	0.204
>7 days	401 (47.57)	101 (25.19)	206 (51.37)	94 (23.44)		

*Note*: Others include commercial insurance, medical aid, and no medical insurance. *p* values of <0.05 are indicated in bold.

Abbreviations: FT, financial toxicity; NA, not available; UEBMI, Urban Employees Basic Medical Insurance; URBMI, Urban and Rural Residents Basic Medical Insurance.

^†^ Medical assistance includes national medical aid, drug donation from enterprises, or participatiion in clinical trials.

### 3.3 Predictors associated with financial toxicity

Table 2 presents the results of univariate and multivariate multinomial logistic regressions. Univariate analyses showed that residence area, residence type, gender, marital status, education experience, work, annual family income, medical insurance, cancer stage, and self‐reported health status were associated with the degree of FT (*p* < 0.05). Multivariate analysis demonstrated that patients living in the western region had 1.95 times (95% CI: 1.18–3.21) the odds of having mild FT and 2.16 times (95% CI: 1.19–3.93) the odds of having moderate and severe FT. Male patients had approximately twice the odds of having mild (*OR* = 2.07, 95% CI: 1.39–3.08) or moderate and severe (*OR* = 1.87, 95% CI: 1.15–3.03) FT compared with that female patients. Patients with a high school education had 2.04 times (95% CI: 1.00–4.18) the odds of having moderate and severe FT compared to those with college‐going experience. Patients with advanced stage cancer trended to >2 times the odds of having mild (*OR*_2 vs. 0–1_ = 2.15, 95% CI: 1.01–4.61), moderate and severe (*OR*_4 vs. 0–1_ = 2.36, 95% CI: 1.15–4.85; *OR*_unknow vs. 0–1_ = 2.37, 95% CI: 1.13–4.94) FT compared to those with early stage cancer.

**TABLE 2:** Multinomial logistic regression of FT

Patient characteristics	Mild versus no FT		Moderate and severe versus no FT	
	Unadjusted *OR* (95% CI)	Adjusted *OR* (95% CI)	Unadjusted *OR* (95% CI)	Adjusted *OR* (95% CI)
Demographic characteristics				
Residence region				
Eastern	ref	ref	ref	ref
Central	1.47 (1.00, 2.15)*	1.31 (0.85, 2.00)	1.52 (0.95, 2.42)	1.34 (0.79, 2.28)
Western	2.18 (1.38, 3.43)***	1.95 (1.18, 3.21)**	2.55 (1.51, 4.31)***	2.16 (1.19, 3.93)*
Residence type				
Rural	ref	ref	ref	ref
Urban	0.59 (0.41, 0.84)**	0.73 (0.43, 1.26)	0.39 (0.25, 0.58)**	0.88 (0.45, 1.70)
Gender				
Female	ref	ref	ref	ref
Male	1.87 (1.32, 2.65)***	2.07 (1.39, 3.08)***	1.50 (0.99, 2.27)	1.87 (1.15, 3.03)*
Age (years)				
<55	ref	ref	ref	ref
55–65	1.06 (0.71, 1.59)	0.86 (0.53, 1.41)	0.83 (0.52, 1.32)	0.55 (0.31, 0.98)*
>65	1.11 (0.71, 1.73)	1.09 (061, 1.96)	0.61 (0.36, 1.06)	0.52 (0.26, 1.06)
Marital status				
Married	ref	ref	ref	ref
Single/divorced/widowed	1.40 (0.62, 3.14)	1.75 (0.71, 4.27)	2.43 (1.03, 5.74)*	2.48 (0.91, 6.77)
Education				
College	ref	ref	ref	ref
High school	1.72 (1.09, 2.72)*	1.41 (0.81, 2.46)	2.99 (1.68, 5.32)***	2.04 (1.00, 4.18)*
Junior school	1.94 (1.24, 3.03)**	1.30 (0.72, 2.34)	2.42 (1.35, 4.32)**	1.00 (0.46, 2.19)
Primary school or less	2.14 (1.28, 3.60)**	1.38 (0.68, 2.81)	3.14 (1.65, 5.96)***	1.10 (0.45, 2.71)
Economic characteristics				
Work				
Public institutions	ref	ref	ref	ref
Private institutions or individuals	1.24 (0.72, 2.15)	0.91 (0.48, 1.73)	1.43 (0.70, 2.89)	0.83 (0.36, 1.92)
Farmer	1.48 (0.89, 2.48)	0.50 (0.23, 1.11)	3.19 (1.71, 5.97)***	1.01 (0.38, 2.64)
Retired	0.93 (0.58, 1.49)	0.63 (0.35, 1.13)	0.96 (0.50, 1.81)	0.70 (0.32, 1.52)
Unemployed	2.41 (1.03, 5.61)*	1.13 (0.42, 3.04)	3.78 (1.43, 9.96)**	1.26 (0.40, 3.98)
Other	0.82 (0.28, 2.42)	0.62 (0.18, 2.15)	1.68 (0.49, 5.78)	1.00 (0.22, 4.53)
Annual family income (1000 yuan)				
<30	ref	ref	ref	ref
30–60	0.90 (0.54, 1.50)	0.90 (0.52, 1.55)	0.67 (0.38, 1.16)	0.89 (0.48, 1.63)
>60	0.28 (0.17, 0.43)***	0.28 (0.16, 0.48)***	0.10 (0.06, 0.18)***	0.14 (0.07, 0.27)***
Medical insurance^†^				
UEBMI	ref	ref	ref	ref
URBMI	1.28 (0.90, 1.83)	1.02 (0.63, 1.67)	2.28 (1.47, 3.55)***	1.45 (0.77, 2.71)
Other	0.34 (0.18, 0.64)***	0.36 (0.18, 0.71)**	0.51 (0.22, 1.18)	0.53 (0.20, 1.37)
Medical assistance				
Yes	0.98 (0.59, 1.62)	0.73 (0.41, 1.29)	1.22 (0.68, 2.17)	0.90 (0.46, 1.78)
No	ref	ref	ref	ref
Clinical characteristics				
Cancer duration (years)				
<1	ref	ref	ref	ref
1–2	1.25 (0.82, 1.91)	1.03 (0.65, 1.65)	1.23 (0.75, 2.02)	0.99 (0.57, 1.74)
≥2	1.28 (0.86, 1.91)	1.24 (0.79, 1.93)	1.10 (0.68, 1.78)	1.15 (0.67, 1.99)
Cancer stage				
0–1	ref	ref	ref	ref
2	2.36 (1.18, 4.71)*	2.15 (1.01, 4.61)*	1.56 (0.60, 4.05)	1.49 (0.51, 4.29)
3	1.66 (0.97, 2.81)	1.43 (0.78, 2.61)	2.31 (1.16, 4.57)*	2.09 (0.95, 4.59)
4	1.73 (1.06, 2.81)*	1.36 (0.78, 2.36)	3.07 (1.65, 5.73)***	2.36 (1.15, 4.85)*
NA	1.30 (0.79, 2.14)	1.20 (0.68, 2.11)	2.36 (1.25, 4.48)**	2.37 (1.13, 4.94)*
Self‐reported health status				
Worse	ref	ref	ref	ref
Moderate	0.70 (0.46, 1.07)	0.79 (0.50, 1.26)	0.42 (0.26, 0.68)***	0.47 (0.27, 0.81)**
Better	0.53 (0.34, 0.82)**	0.50 (0.31, 0.82)**	0.28 (0.16, 0.48)***	0.24 (0.13, 0.43)***
Length of stay				
≦7 days	ref	ref	ref	ref
>7 days	0.75 (0.54, 1.05)	0.64 (0.44, 0.94)*	0.90 (0.60, 1.35)	0.75 (0.47, 1.19)

*Note*: Others include commercial insurance, medical aid, and no medical insurance.

Abbreviations: CI, confidence interval; FT, financial toxicity; NA, not available; OR, odds ratio; ref, reference; UEBMI, Urban Employees Basic Medical Insurance; URBMI, Urban and Rural Residents Basic Medical Insurance.

^†^ Medical assistance includes national medical aid, drug donation from enterprises, or participation in clinical trials.

* *p* < 0.05.

** *p* < 0.01.

*** *p* < 0.001.

Higher annual family income and better self‐reported health status proved to be protective predictors of FT. Patients with an annual family income of over 60,000 yuan had 72% lower odds of having mild FT (*OR* = 0.28, 95% CI: 0.16–0.48), and 86% lower odds of having moderate and severe FT (*OR* = 0.14, 95% CI: 0.07–0.27) compared to those with an annual family income below 30,000 yuan. Patients with a self‐reported health status of “better” than peers had 50% lower odds of having mild FT (*OR* = 0.50, 95% CI: 0.31–0.82) and 76% lower odds of having moderate and severe FT (*OR* = 0.24, 95% CI: 0.13–0.43); those with “moderate” health had 53% lower odds of having moderate and severe FT (*OR* = 0.47, 95% CI: 0.27–0.81) compared to those with “worse” health. Patients aged 55–65 years had 45% lower odds (*OR* = 0.55, 95% CI: 0.31–0.98) for moderate and severe FT compared with those aged <55 years. Patients with a length of stay over 7 days had 36% lower odds (*OR* = 0.64, 95% CI: 0.44–0.94) for mild FT compared with that of their counterparts (Table 2).

### 3.4 Patient coping actions

**FIGURE 2:**
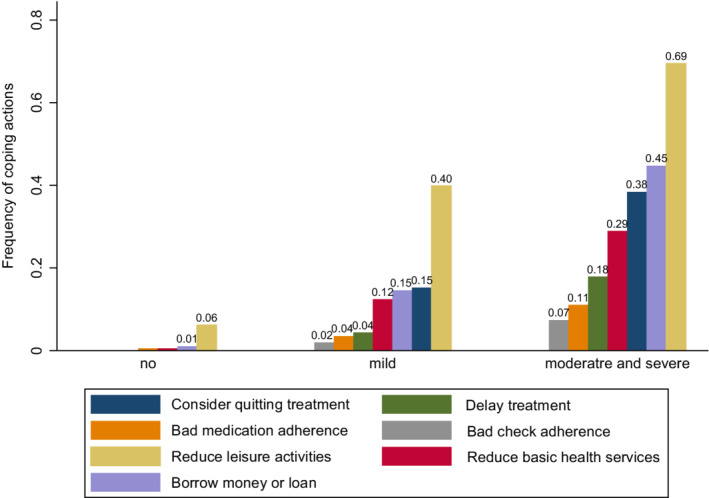
Frequency of patient coping actions by a degree of financial toxicity

The most common coping actions included reducing expenditures on leisure activities, borrowing money, and considering quitting treatment (Figure 2). Approximately 69% of patients with moderate and severe FT reduced their expenditures on leisure activities, nearly 45% borrowed money, and 38% considered quitting treatment. Relative to those with lower FT, patients with higher FT tended to have a lower level of medical compliance, including medication adherence, check adherence, and delayed treatment as well as reduced living expenditures on leisure activities and basic health services (all *P*_trend_ <0.001).

## 4 DISCUSSION

Over the past decade, FT has become a major concern when making decisions regarding clinical treatment. This study uses a validated tool to measure FT and is the first national survey in a population of patients with lung cancer in China. Our results demonstrate that three out of four (76.99%) patients with lung cancer had FT and nearly one out of four had moderate and severe FT (mild FT: 54.45%; moderate and severe FT: 22.54%). Living in the western region of China, being male, having a lower educational level, and having advanced‐stage lung cancer were all strongly associated with higher FT. Higher annual family income and better self‐reported health status were strongly associated with lower FT.

### 4.1 The level of FT of patients with lung cancer in China

The prevalence of FT in patients with lung cancer in China was relatively high, with a prevalence rate of over 75% and a median score of 20. One study in the United States found that only approximately 50% of patients with stages 2–4 lung cancer had FT.^24^ Another study in Canada demonstrated the median score of FT was 21 in patients with stages 3–4 lung cancer, which is higher than that the median score of 20 in our study, indicating a lower financial burden.^12^ Only one study conducted in the western region of China did not report the scores or prevalence of FT in lung cancer patients.^25^ Additionally, patients with lung cancer in China and worldwide had higher FT compared with that for other cancer types, such as breast, bladder, and localized prostate cancers.^6^, ^33^, ^34^ Although Universal Healthcare Coverage has existed in China since 2009, the proportion of out‐of‐pocket costs in total health expenditure is more than 25%.^35^ Furthermore, with the development of innovative treatments for lung cancer such as immune and targeted therapy, treatment‐related costs continue to increase.^36^, ^37^ Overall, a relatively high prevalence of FT in patients with lung cancer in China presents a serious challenge, with urgent attention and interventions needed.

### 4.2 Predictors associated with FT

Socioeconomic status (SES) was a strong predictor of FT. This study demonstrated that lower education level and annual family income were strong predictors of higher FT. Previous studies have shown similar results regardless of the cancer type.^38^, ^39^ Families or individuals with a low income usually cannot financially afford to be afflicted with serious diseases. Additionally, farmers and unemployment were associated with more severe FT in univariable analysis. This indicated that farmers and unemployed individuals were vulnerable to FT, although the relationship was not statistically significant when income and education were controlled. Being a farmer and unemployed yielded lower SES, lower savings and income, worse insurance coverage, and tended to face higher economic stress when they are ill. Men who are socially accepted as the main breadwinner, tend to greatly influence annual family income due to loss of productivity and tend to feel higher stress in times of illness.^40^ Furthermore, the western region of China is less developed and residents usually have less income and savings.^41^

Regarding clinical factors, advanced stage and worse self‐reported health status were associated with higher FT. Patients with advanced stage or worse self‐reported health status require more complex treatment schemes and pay more. Additionally, the complex treatment as a result of multiple comorbidities restricts patients from returning to work, resulting in greater FT.^42^ Overall, low income, low savings, and high costs related to treatments were the main causes of patients falling into the financial burden and enormous psychological stress. In China, improvement in health policies is needed to relieve this situation in the future through broader coverage of social medical insurance, more inclusions of expensive targeted drugs and testing, as well as more medical aid from the government, society, and individuals.

### 4.3 Patient coping actions

Patient coping actions in response to FT lead to adverse consequences such as worse quality of life and reduced medical compliance.^43^, ^44^ Our study decategorized coping actions into seven items from two main domains. These domains were debts and reducing living expenditures and medical compliance, including considering quitting treatment, delayed treatment, bad medication adherence, and check adherence. Patients tended to use coping actions of the first domain, accounting for 30%–70% of patients with moderate and severe FT. The medical compliance domain, which is related to direct negative consequences, accounted for a large proportion of patients with deteriorating FT. These alert for specific attention to patients with high financial risks.

### 4.4 Strengths and limitations

This study estimated the prevalence and degree of FT in a large sample with a diversity of geographic locations and multicenter collaborations in China. Our results can help identify patients vulnerable to FT and find the relationship between FT and patient coping actions, reflecting an urgent need for interventions in this population.

This study has some limitations. Although we sampled patients across the country, our study sites were at tertiary cancer hospitals, which represented mostly patients with ongoing diseases and treatment courses. This limits the generalization of our study results to the general population. Additionally, information on the stage and duration of disease were self‐reported by patients, which may have led to recall bias. The magnitude of the bias may be small as the patients were interviewed in hospitals and were close to discharge. Finally, the nature of the cross‐sectional design limits the power to determine a causal relationship in our study.

## 5 CONCLUSIONS

In this national survey, a relatively high prevalence of FT was observed in patients with lung cancer in China despite remarkable progress made in the past two decades with regard to Universal Healthcare Coverage. This presents a serious challenge primarily to patients with lung cancer. More severe FT was observed in patients living in the less‐developed western region of China, males, patients with lower educational levels, lower annual family incomes, advanced stages of lung cancer, and worse health status. Patients with moderate and severe FT are more likely to use coping actions, including medical noncompliance, incurring medical debts, and reducing living expenditures. Keen attention must be paid to reducing the disproportionate high financial risks of those patients with low SES.

## AUTHOR CONTRIBUTIONS

**Meicen Liu:** Conceptualization (equal); data curation (lead); formal analysis (lead); funding acquisition (supporting); investigation (equal); methodology (equal); project administration (supporting); software (lead); validation (equal); visualization (lead); writing – original draft (lead); writing – review and editing (equal). **Dengmin Huang:** Conceptualization (supporting); data curation (supporting); formal analysis (supporting); methodology (equal); supervision (equal); writing – review and editing (supporting). **Yuanli Liu:** Conceptualization (lead); investigation (lead); methodology (equal); project administration (lead); resources (lead); validation (equal); writing – review and editing (lead).

## FUNDING INFORMATION

This work was supported by the Chinese Academy of Medical Sciences (grant number 2021‐I2M‐1‐046).

## CONFLICT OF INTEREST

The authors have no relevant financial or nonfinancial interests to disclose.

## ETHICS APPROVAL

This study was performed in line with the principles of the Declaration of Helsinki. Approval was granted by the Ethics Committee of Institute of Medical Biology of Chinese Academy of Medical Sciences (IPB‐2020‐23).

## CONSENT TO PARTICIPATE

Informed consent was obtained from all individual participants included in the study.

## Data Availability

The data sets are available from the corresponding author upon reasonable request.
